# Correlation Between T_1_ Precipitation and Strength–Corrosion Performance in 2060 Al–Li Alloy

**DOI:** 10.3390/ma19122598

**Published:** 2026-06-16

**Authors:** Juan Yu, Zhaohui Feng, Guoai Li, Quanyi Xue, Sai Tang

**Affiliations:** 1 Beijing Engineering Research Center of Advanced Aluminum Alloys and Applications, Beijing Institute of Aeronautical Materials, Beijing 100095, China; yuer1437@126.com (J.Y.); yuzhao_0210@163.com (Z.F.); liguoai_1@163.com (G.L.); 2 National Key Laboratory of Science and Technology on High-Strength Structural Materials, Central South University, Changsha 410083, China; s.tang@csu.edu.cn

**Keywords:** 2060 Al–Li alloy, T8 aging treatment, T1 precipitation, mechanical properties, intergranular corrosion

## Abstract

**Highlights:**

- Optimal T8 treatment at 150 °C/48 h yields YS of 521 MPa, elongation of 11.1%, and shifts corrosion mode from IGC to pitting.
- With varying aging temperatures, the degree to which the mechanical anisotropy of the material changes with aging time differs.
- Continuous-to-discontinuous transition of grain-boundary T_1_ morphology dictates the intergranular corrosion (IGC) → pitting → IGC evolution.

**Abstract:**

This study aims to identify the optimal aging regime that balances strength and intergranular corrosion (IGC) resistance in a 2060 Al–Li alloy under T8 temper. The evolution of microstructure, mechanical properties, and IGC behavior was systematically investigated across various aging conditions. The most relevant results show that the optimal regime for the 3% pre-stretched alloy is 150 °C for 32–48 h. At the peak-aged state (150 °C/48 h), the alloy achieves a yield strength (YS) of 521 MPa and ultimate tensile strength (UTS) of 541 MPa in the longitudinal (L) direction, and 486 MPa and 548 MPa in the long-transverse (LT) direction, with elongations of 11.1% and 12.2%, respectively. Under this condition, the corrosion mode shifts from IGC to pitting, with a maximum pitting depth of 98.6 μm. Microstructural analyses confirm that the T_1_ (Al_2_CuLi) phase is the primary strengthening precipitate. Critically, as aging temperature and time increase, T_1_ plates extensively nucleate and grow within grain interiors, while their distribution at grain boundaries (GBs) becomes discontinuous. This discontinuous GB precipitate morphology interrupts continuous anodic dissolution channels, thereby significantly enhancing localized corrosion resistance. Notably, these findings can offer practical guidance for industrial heat treatments of third-generation Al–Li alloys, particularly for safety-critical aerospace components where both strength and corrosion resistance are mandatory.

## 1. Introduction

The energy crisis of the 1970s catalyzed a paradigm shift in the aviation industry, necessitating the aggressive development of lightweight structural materials. Concurrently, the rapid ascent of carbon fiber-reinforced composites posed a significant competitive challenge to traditional aeronautical aluminum alloys. In response, aluminum–lithium (Al–Li) alloys emerged as a critical solution due to their low density and high elastic modulus [1,2,3,4,5]. For each addition of 1 wt.% of Li in the aluminum matrix, the density decreases by 3%, and the elastic modulus increases by 6% [6,7,8]. By substituting conventional aluminum alloys with Al-Li variants in load-bearing components, aircraft weight can be reduced by 8–15%, while structural stiffness is enhanced by 15–20% [9].

As of the current technological landscape, third-generation Al–Li alloys represent the state-of-the-art in aerospace applications [10,11,12,13]. A prominent example is the 2060 Al–Li alloy, registered by Alcoa in 2011 [2,11]. This alloy has a nominal composition of Al-(3.5–4.5)Cu-(0.6–1.0)Li-(0.3–0.8)Mg-(0.2–0.6)Ag-(0.1–0.5)Zn with minor Zr and Mn additions; it exhibits low density (~2.70 g/cm^3^), high modulus (~79 GPa), and typical T8 temper strength >500 MPa [11,14]. It is characterized by reduced anisotropy, superior strength–ductility synergy, and exceptional thermal stability. It was designed to replace heritage alloys such as AA2524-T3vc and 2024-T351, offering potential weight savings of 7% and 14% [15], respectively. Notably, the 2060 alloy has already seen successful industrial integration in the fuselage sections of the COMAC C919 narrow-body aircraft [16,17,18]. Although it has been widely used in industry, a systematic understanding of its long-term performance under various aging regimes is still lacking.

Belonging to the Al–Cu–Li system, the 2060 alloy leverages an optimized Cu/Li ratio and multi-component microalloying to achieve high static strength—particularly YS—and fracture toughness without compromising fatigue resistance [19,20,21,22]. The existence of different precipitates and their interactions with dislocations plays a pivotal role in the mechanical performance [23,24,25]. Despite its industrial adoption, fundamental research into its long-term performance remains fragmented [26,27]. Existing studies have predominantly focused on weldability and manufacturing processes, leaving a significant gap in the systematic understanding of its mechanical behavior and corrosion resistance [28,29,30,31]. Recent studies by Liu et al. [32] on high-Mg 2060 variants aged at 155 °C identified a complex precipitation sequence involving θ′, T1, and S′ phases, with the T1 phase acting as the primary strengthening agent. Furthermore, Jia et al. [33] investigated the interplay between cryogenic processing and secondary solution annealing, reporting an optimal YS of 494 MPa and 6% elongation after 35 h of aging at 165 °C. Despite these insights, no prior work has systematically addressed how the coupled evolution of T1 and θ′ precipitates under diverse aging regimes (particularly T8 temper, i.e., cold work followed by artificial aging) influences the strength–toughness–corrosion trade-off in the 2060 alloy. In particular, how the evolution of T1 and θ′ phases under different thermal histories simultaneously dictates the strength–toughness trade-off and the localized corrosion susceptibility remains poorly understood.

This study aims to bridge this gap by conducting a systematic investigation into the microstructural evolution of the 2060 Al–Li alloy under diverse aging regimes. By elucidating the mechanisms governing strength–toughness trade-offs and corrosion susceptibility, this work provides both theoretical and practical insights for the microstructural design and manufacturing optimization of high-performance Al–Cu–Li alloys.

## 2. Materials and Methods

The raw material for this study was a 2.0 mm thick 2060-T8E30 Al–Cu–Li alloy plate manufactured by Southwest Aluminum Co., Ltd. (Chongqing, China). The detailed chemical composition of the alloy is summarized in Table 1. It should be noted that this alloy can be classified as an Al–Cu alloy from the point of compositional majority; however, to highlight the impact of Li-containing precipitates on material properties, this study refers to it as an Al–Li alloy. To investigate the effects of precipitation kinetics, the as-received plates were first subjected to a solution treatment at 500 °C for 30 min in a salt bath furnace, followed by immediate water quenching to room temperature to obtain a supersaturated solid solution (SSS). Subsequently, the quenched samples were pre-stretched to a constant strain of 3% along the rolling direction (L direction) to introduce density-controlled dislocations, which help to overcome the energy barrier for precipitation and serve as heterogeneous nucleation sites for T_1_ precipitates during subsequent artificial aging, thereby accelerating the age-hardening response and promoting a fine, uniform precipitate distribution. Finally, artificial aging treatments were conducted in a circulating hot-air furnace across a temperature range of 120 °C to 150 °C for durations spanning 0 to 96 h. This processing route was designed to achieve various aging states (under-aged, peak-aged, and over-aged) and to systematically evaluate the microstructural evolution of the T8 temper. It is noted that the T8 temper involves solution treatment, cold work (2–6%), and then aging; T1 (Al_2_CuLi) and θ′ (Al_2_Cu) are the key strengthening precipitates.

**Figure 1:**
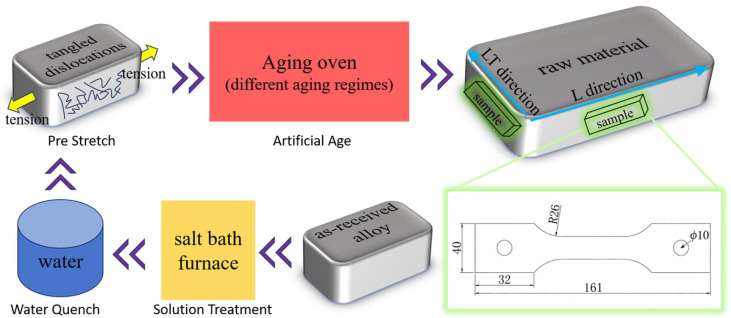
Diagram showing the preparation process of the material, including the sample size for tensile tests, whose unit is mm.

Uniaxial tensile tests were performed at room temperature using an INSTRON universal testing machine (Instron, Norwood, MA, USA). To ensure quasi-static conditions, a constant strain rate of 2.5 × 10^−4^ s^−1^ was maintained throughout the tests. Tensile specimens were machined according to the geometry illustrated in Figure 1, with a thickness consistent with that of the raw material (i.e., 2 mm). The longitudinal axes were arranged respectively along the L direction (rolling direction) and the LT direction (long-transverse direction) to evaluate mechanical anisotropy. To ensure statistical reliability and reproducibility, at least three specimens were tested for each heat treatment condition.

The Vickers hardness was measured using a universal hardness tester, with the average value calculated from at least five indentations to ensure statistical accuracy. Electrical conductivity was determined using a Sigma test 2.069 eddy current conductometer (Sigma, Fukushima, Japan). IGC tests were conducted in accordance with the ASTM G110 standard [34]. The test surfaces were selected from the rolling plane (L-LT), followed by mechanical grinding, polishing, and degreasing with acetone. After rinsing with distilled water, the specimens were immersed in a corrosive solution consisting of 57 g/L NaCl and 10 mL/L H_2_O_2_ at 35 pm 2 °C for 6 h. Post-corrosion morphology and cross-sectional attack depths were characterized using an optical microscope (OM).

The precipitates were investigated using a JEM-2000FX transmission electron microscope (TEM) (JEOL, Tokyo, Japan). Thin foils for TEM observation were prepared by initially grinding the samples to a thickness of approximately 50 μm. Final thinning was performed via twin-jet electropolishing at −25 °C and a dual-voltage of 15 V. The electrolyte solution consisted of 30% HNO_3_ and 70% CH_3_OH.

## 3. Results

**Figure 2:**
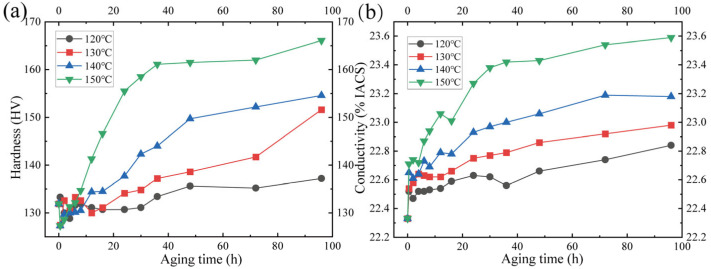
(**a**) Hardness and (**b**) conductivity of 2060 alloy after different aging processes. The gray circle, red rectangle, blue triangle and green triangle, respectively, represent aging temperatures of 120, 130, 140 and 150 °C.

Figure 2 illustrates the evolution of hardness and electrical conductivity for the 2060 Al–Li alloy as a function of aging time (0–96 h) at temperatures ranging from 120 °C to 150 °C. As shown in Figure 2a, the alloy exhibits a relatively stable hardness profile during the initial 8 h of aging across all temperatures, suggesting a latent period for significant precipitate nucleation. Beyond this stage, the age-hardening response becomes highly temperature-dependent. At 120 °C, the hardness shows only a marginal increase, rising from an initial value of 131.9 HV to 137.2 HV after 96 h, indicating sluggish precipitation kinetics at lower temperatures. A similar but more pronounced trend is observed at 130 °C, where the hardness steadily increases to 151.6 HV after 72 h of aging. In contrast, at higher aging temperatures of 140 °C and 150 °C, the alloy demonstrates a rapid hardening rate during the first 40 h. Subsequently, the hardness curves reach a plateau, representing the peak-aged (PA) state. Specifically, the hardness reaches 149.7 HV after 48 h at 140 °C and a maximum of 161.5 HV after only 36 h at 150 °C. These results indicate that increasing the aging temperature significantly accelerates the precipitation of strengthening phases (such as T_1_, and θ′), leading to a higher peak hardness and a shorter time to reach the T8 state.

The evolution of electrical conductivity for the 2060 Al–Li alloy is presented in Figure 2b. The data indicates a monotonic increase in conductivity with both increasing aging temperature and prolonged aging time, a phenomenon primarily attributed to the reduction in electron scattering as solute atoms (Cu, Li, and Mg) precipitate from the SSS. During the initial aging stage (t < 4 h), a rapid surge in electrical conductivity is observed across all temperatures, corresponding to the initial decomposition of the SSS and the formation of Guinier–Preston (GP) zones or precursor phases. As aging progresses, the samples treated at 120 °C and 130 °C exhibit a significantly lower rate of increase, suggesting a slow solute-depletion process.

In contrast, the conductivity of samples aged at 140 °C and 150 °C continues to rise sharply until approximately 36 h, after which it enters a plateau phase, remaining nearly constant up to 96 h. Specifically, after 36 h of aging, the conductivity reaches 23.0% IACS (International annealed copper standard) and 23.42% IACS at 140 °C and 150 °C, respectively. This plateau in conductivity correlates well with the hardness trends observed in Figure 2a, signaling that the solute concentration in the matrix has reached a near-equilibrium state corresponding to the peak-aged (PA) microstructure.

**Figure 3:**
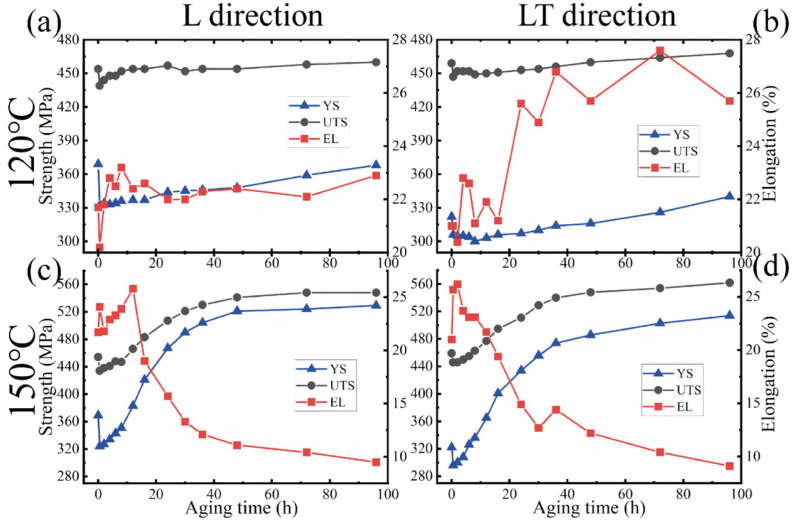
Tensile properties of the 2060 alloy with different aging processes: (**a**) L direction, 120 °C; (**b**) LT direction, 120 °C; (**c**) L direction, 150 °C; (**d**) LT direction, 150 °C. Within the figures, the blue triangle, the gray circle and the red rectangle respectively represent the yield strength, ultimate tensile strength and elongation.

The evolution of tensile properties for the 2060 Al–Li alloy aged at 120 °C and 150 °C is illustrated in Figure 3. At the aging temperature of 120 °C, both the YS and UTS exhibit a slight initial decrease within the first 0.5 h, followed by a marginal increase as aging time progresses. Conversely, the elongation (EL) shows a gradual improvement, particularly for specimens oriented in the LT direction.

At 150 °C, the alloy demonstrates a more dynamic mechanical response. A sharp drop in strength is observed during the earliest stages of aging, likely associated with the recovery of the pre-stretched microstructure, followed by a rapid increase driven by the accelerated precipitation of strengthening phases. Correspondingly, the elongation initially increases before gradually declining as the alloy approaches the peak-aged state. Beyond 48 h of aging at 150 °C, the tensile properties stabilize, reaching a plateau that persists up to 96 h.

Notably, the time-dependent evolution of mechanical properties follows a consistent trend for both L and LT orientations, though a degree of anisotropy in strength and ductility remains evident. Considering both mechanical performance and processing efficiency, the 150 °C for 48 h regime is identified as the optimal aging treatment. Under these conditions, the L-direction specimens achieve a YS of 521 MPa, an UTS of 541 MPa, and an EL of 11.1%. Similarly, the LT-direction specimens exhibit a YS of 486 MPa, an UTS of 548 MPa, and an EL of 12.2%.

**Figure 4:**
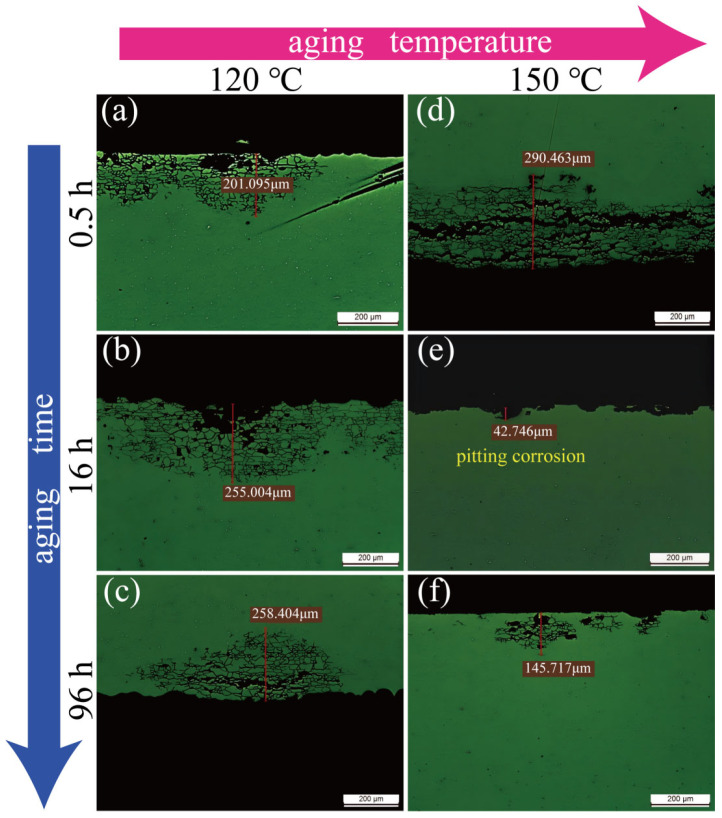
Intergranular corrosion morphologies of 2060 alloy at different aging processes obtained from OM: (**a**) 120 °C, 0.5 h; (**b**) 120 °C, 16 h; (**c**) 120 °C, 96 h; (**d**) 150 °C, 0.5 h; (**e**) 150 °C, 16 h; (**f**) 150 °C, 96 h.

**Figure 5:**
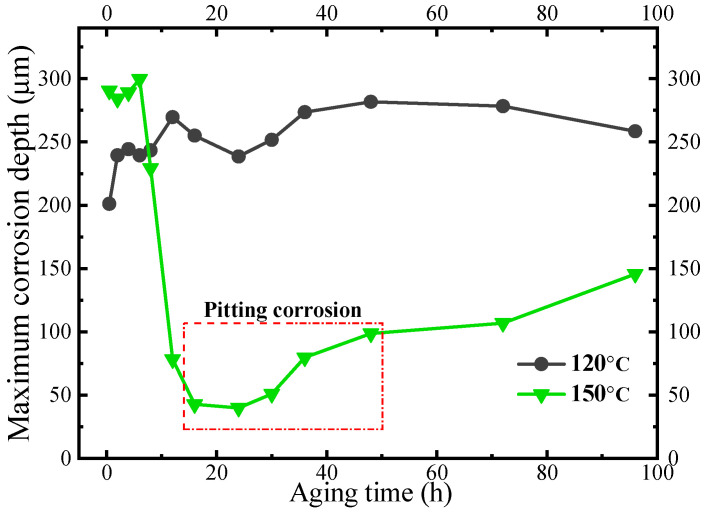
Maximum intergranular corrosion depths of the 2060 alloy as a function of aging time at 120 °C (gray circles) and 150 °C (green triangles).

Figure 4 presents the evolution of the corrosion morphology for the 2060 Al–Li alloy aged at 120 °C and 150 °C, with the corresponding maximum corrosion depths summarized in Figure 5. For the specimens aged at 120 °C, IGC is the predominant corrosion mode across all aging durations. The maximum IGC depth exhibits minor fluctuations, ranging between 201.0 and 281.7 μm, suggesting that the lower aging temperature does not significantly alter the susceptibility of the GBs to localized attack within the investigated timeframe. In contrast, the corrosion behavior at 150 °C displays a distinct transition in both morphology and severity. The maximum corrosion depth decreases sharply from 290.4 μm (initial stage) to a minimum of 39.8 μm after 24 h of aging. Within the aging window of 16 to 48 h, the corrosion mechanism shifts from IGC to pitting corrosion, characterized by relatively shallow pit depths. Specifically, the maximum pitting depth increases from 42.7 μm at 16 h to 98.6 μm at 48 h.

However, as the aging time exceeds 48 h (entering the over-aged regime), the corrosion mode reverts to intergranular corrosion, and the attacked depth gradually increases with further aging. This “U-shaped” trend in corrosion susceptibility at 150 °C is likely governed by the continuous precipitation and coarsening of the T_1_ (Al_2_CuLi) phase at the GBs and the concomitant development of precipitate-free zones (PFZs). In the over-aged condition, the wide PFZs are expected to be more anodic than the matrix based on well-established literature about aluminum alloys [35,36], while the coarsened T_1_ particles remain anodic as well. The resulting spatial distribution of anodic regions may reestablish a continuous dissolution path along the GBs, leading to the reappearance of IGC, which alters the electrochemical potential difference between the grain interior and the boundary [37,38].

**Figure 6:**
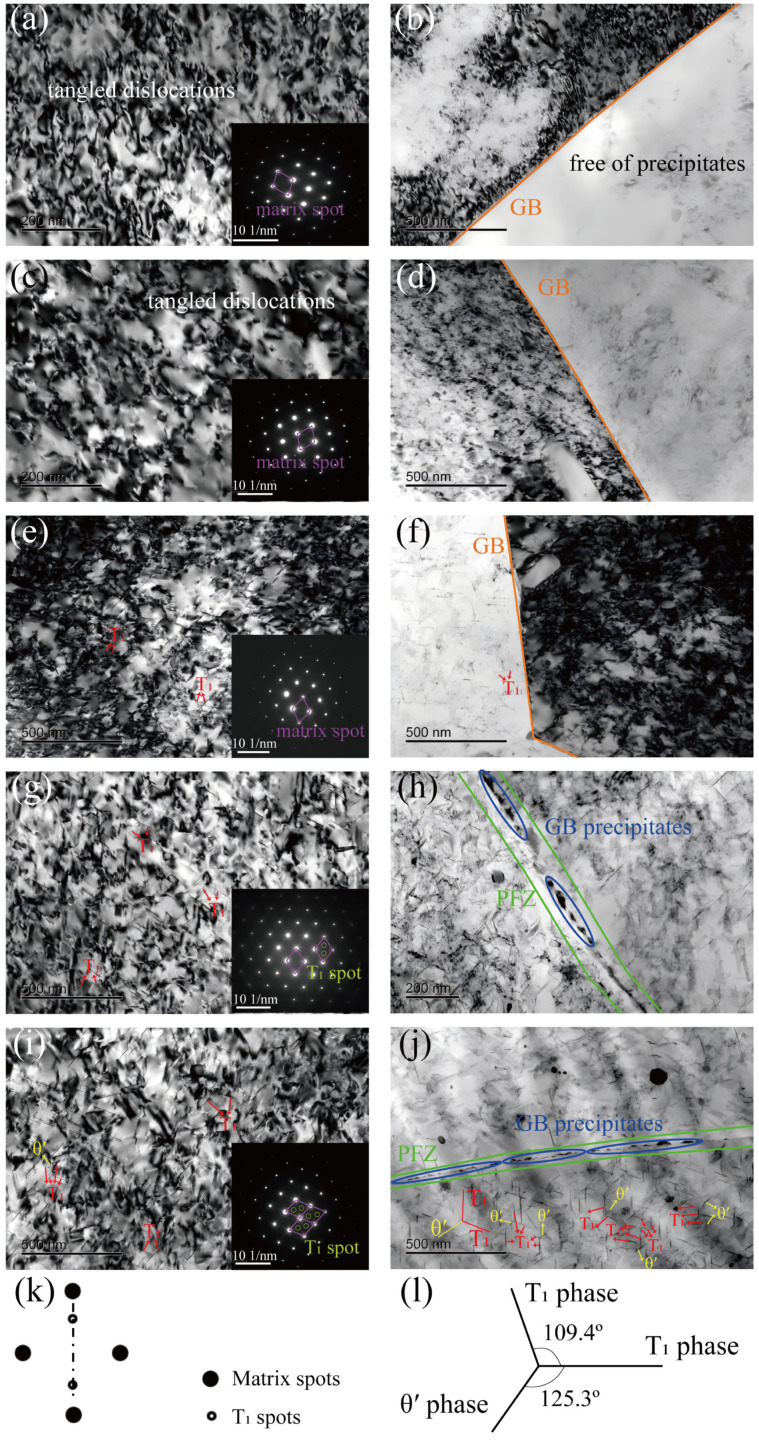
TEM images of the 2060 alloy aged at varied times and temperatures: (**a**,**b**) 0 h; (**c**,**d**) 120 °C, 96 h; (**e**,**f**) 150 °C, 36 h; (**g**,**h**) 150 °C, 48 h; (**i**,**j**) 150 °C, 96 h. There are plenty of tangled dislocations in the materials due to the pre-stretching, and their number decreases with the increase in aging time and temperature. The inset figures represent the SAED pattern of the matrix and the T_1_ phase, with the zone axis in the [110]_FCC_ direction; (**k**) a schematic of the SAED pattern of the matrix and the T_1_ phase; (**l**) a schematic of the orientation relationship between T_1_ and θ′ phases.

Figure 6 displays the TEM micrographs and corresponding selected area electron diffraction (SAED) patterns for the 2060 Al–Li alloy under various aging conditions. In the as-quenched state and after aging at 120 °C for 96 h, a high density of tangled dislocations are observed within the grain interiors, as shown in Figure 6a,c. This is attributed to the 3% pre-stretching process performed prior to the artificial aging. At this low temperature, a distinct PFZ is already visible along the GBs (Figure 6b,d), while the matrix remains largely devoid of observable precipitates, confirming the sluggish precipitation kinetics at 120 °C.

**Figure A1:**
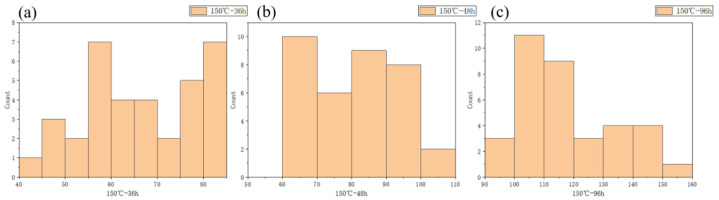
Histogram showing the distribution of the size of 35 T1 phases under different aging times; this distribution was calculated based on 3 TEM images for each condition: (**a**) for 36 h; (**b**) for 48 h; (**c**) for 96 h.

In contrast, aging at 150 °C triggers significant microstructural transformations. After 36 h, the dislocation density decreases slightly due to recovery, and the nucleation of fine T_1_ plates (approximately 66.4 nm in average length, and statistical results are recorded in Figure A1) is observed, primarily pinned to the dislocation lines. The presence of these precipitates is confirmed by the characteristic streaks and spots in the SAED patterns (Figure 6e,k). Meanwhile, the precipitates initially form a continuous chain-like morphology at the GB (Figure 6f).

As the aging time increases to 48 h (peak-aged state), the T_1_ precipitates undergo substantial growth, reaching an average length of 80.2 nm. The SAED pattern in Figure 6g shows intense diffraction spots corresponding to the T_1_ phase, indicating a high volume fraction of this strengthening agent. Notably, the GB morphology evolves from a continuous strip to a discontinuous distribution of coarse particles (Figure 6h), which is likely responsible for the improved intergranular corrosion resistance observed in the previous sections. Upon reaching 96 h (over-aged state), the number of tangled dislocations decreases significantly, and the T_1_ plates further coarsen to approximately 117.8 nm in average length. At the same time, the increase in aging time contributes to the appearance of θ′ phases shown in Figure 6i,j, which is confirmed with the help of Figure 6k,l. θ′ precipitates appear as thin plates lying on {100} Al planes, with a crystallographic orientation relationship of (100)θ′//(100)Al and [001]θ′//[001]Al, and the relative orientation of T1 and θ′ follows the rule displayed in Figure 6l. Compared to the dominant T_1_ phase, θ′ is much less abundant and its volume fraction is low. It only appears after prolonged aging, when T_1_ has already coarsened. Therefore, θ′ plays a minor role in the mechanical properties and corrosion behavior of the alloy under the conditions investigated. While it can be seen from Figure 6j that GB precipitates remain discontinuously distributed, the coarsening of the matrix T_1_ phase leads to the plateau or slight decline in hardness and strength as previously documented.

## 4. Discussion

### 4.1. Influence of Aging Regimes on Mechanical Properties

In the 2060 Al–Li alloy, the pre-stretching treatment prior to artificial aging introduces a high density of dislocations. These defects serve as high-energy heterogeneous nucleation sites, effectively lowering the energy barrier for precipitate formation. The three main equilibrium phases in the Al–Cu–Li system are T_1_ (Al2CuLi), θ (Al2Cu) and δ (Al_3_Li) phases, respectively. However, under typical aging temperatures (≤150 °C), precipitation proceeds via metastable intermediates before reaching equilibrium [39]. The nucleation mechanisms differ among these phases. The T_1_ phase is the main strengthening precipitate in this alloy. The strain field around dislocations reduces the critical free energy for T1 nucleation, leading to a fine, dense distribution of T_1_ plates on {111} Al planes. The θ′ phase is the metastable precursor to θ, and it nucleates homogeneously on {100} Al planes, without requiring dislocations. Its nucleation is favored by higher Cu supersaturation and higher temperatures. In this study, θ′ is observed only in the over-aged state (aging at 150 °C for 96 h), with a much lower number density than T_1_. Because of the low Li content and the consumption of Li in T1, no δ is observed in this alloy. Thus, under equilibrium conditions (long aging times at 150 °C), T1 remains the primary precipitate, with minor θ′ appearing in the over-aged state. No transformation to fully equilibrium θ or δ occurs within the aging window mentioned in this work. Thermodynamically, the nucleation and growth kinetics of the strengthening phases are primarily driven by the aging temperature [40,41].

At a relatively low temperature of 120 °C, the thermal activation energy is insufficient to trigger extensive T_1_ precipitation. Consequently, the microstructure remains dominated by the dislocation substructure, resulting in negligible changes in hardness and tensile strength even with prolonged aging. As the temperature increases to 130 °C, the T_1_ phase begins to nucleate; however, the low diffusion rate at this temperature results in a low number density and limited particle size. Such a sparse distribution of fine precipitates is insufficient to effectively impede dislocation glide or promote coplanar slip bypass, thus offering limited strengthening effects. Compared with other Al–Cu–Li alloys where higher driving forces are required, the prolonged incubation period at 120–130 °C indicates that the chemical driving force at lower temperatures cannot overcome the strain energy barrier for T_1_ nucleation on dislocations [42].

In contrast, at 140 °C and 150 °C, the increased thermodynamic driving force significantly accelerates both the nucleation and growth rates of the T_1_ phase. The substantial increase in the volume fraction and dimensions of the T_1_ plates provides effective obstacles to dislocation motion, leading to a marked enhancement in YS through the Orowan strengthening mechanism [43]. Beyond 48 h at 150 °C, the precipitation process approaches an equilibrium state where the growth rate of T_1_ stabilizes, leading to the observed plateau in mechanical properties. That is to say, mechanical properties like YS, UTS and hardness are mainly controlled by the characteristics of the T_1_ phases in this alloy. And the temperature has a large impact on the nucleation kinetics of T1 phase, leading to varying degrees of the precipitation strengthening mechanism, which demands determining an optimal aging regime for the Al–Cu–Li system.

Additionally, the mechanical anisotropy (higher YS in the L than the LT direction) is linked to the crystallographic alignment of T_1_ plates. In pre-stretched Al–Li plates, dislocations align preferentially along slip planes parallel to the rolling direction, inducing a strong variant selection of the T_1_ phases. During L-direction tensile deformation, these dominant variants experience higher resolved shear stress, maximizing their efficiency in hindering dislocation motion [44].

### 4.2. Correlation Between Aging and Corrosion Behavior

The localized corrosion susceptibility of Al–Cu–Li alloys is fundamentally governed by the electrochemical potential difference between the secondary phases and the aluminum matrix [45,46]. The driving energy for corrosion originates from the galvanic cell formed between precipitates and the Al matrix. In this system, the T_1_ phase acts as an anodic phase, being more susceptible to dissolution in corrosive media. Therefore, the IGC resistance is intrinsically linked to the morphology and distribution of T_1_ precipitates both within the grain interiors and along the GBs. The main reactions that occur within the system are summarized in Table A1.

At 120 °C, the nucleation of precipitates is suppressed, and the high-density residual dislocation networks serve as preferential pathways for corrosive attack, leading to persistent and severe IGC. At the higher temperature of 150 °C, the T1 phase preferentially nucleates at high-energy GBs during the early stages of aging, forming continuous precipitation bands. These bands are inferred to act as sacrificial anodic channels, resulting in severe IGC.

Unlike the case at 120 °C, where the maximum corrosion depth changes within a range of 80.6 μm regardless of the aging time, a U-shaped maximum corrosion depth curve with a range of 259.8 μm occurs at 150 °C with increasing aging time. At the aging time of 16h and before the peak-aged state, T1 plates within grains emerge, consuming the available solute atoms (Cu and Li) and decreasing their continuity along GBs. This interrupts the continuous anodic dissolution paths required for IGC. Meanwhile, the newly formed fine intragranular T1 plates create micro-galvanic cells at many sites, initiating pitting corrosion with shallower corrosion depth, thereby causing a transition from IGC to pitting. Pits initiate at the matrix/T1 interfaces where the passive film is the weakest [45,46].

It should be noted that such a transition agrees with the boundary dissolution theory in Al–Li alloys, where disrupting the spatial continuity of anodic GB precipitates suppresses the intergranular channels [47]. The pre-strain in 2060 accelerates intragranular precipitation kinetics, starving the GBs of solutes earlier to break this continuity.

However, upon further aging (beyond 48 h), the continued coarsening of matrix precipitates leads to the widening of PFZs adjacent to the GBs. It is believed that solute-depleted regions are more anodic than the T_1_-rich matrix/GB regions [45,46], so it is reasonable to infer that a potential gradient develops between the PFZs and others. This gradient is expected to establish a new galvanic cell, which may facilitate the re-emergence of IGC through the formation of wide, continuous dissolution channels. Such IGC recurrence underlines the critical impact of PFZ development. Extended aging at 150 °C broadens the solute-depleted PFZ via solute diffusion toward coarsening GB precipitates. Since the PFZ has a lower solute concentration (Cu, Li) than the T_1_-dense grain interior, it becomes highly anodic, creating a severe micro-galvanic potential gradient that re-establishes continuous dissolution pathways [48].

That is to say, residual dislocations from pre-stretching and precipitates from aging contribute to the mechanical properties and the corrosion behaviors of Al–Li alloys simultaneously. Therefore, considering the balance between mechanical properties and corrosion resistance, the optimal aging window for the 3% pre-stretched 2060 Al–Li alloy is determined from a series of aging regimes.

## 5. Conclusions

This study investigates the effects of aging conditions on the microstructure, mechanical properties, and corrosion resistance of a 2.0 mm thick 2060-T8E30 Al–Li alloy plate. The main conclusions are as follows:

1. Aging temperature controls strengthening. At 120–130 °C, T_1_ nucleation is limited, causing slow hardening. At 140–150 °C, the driving force for T_1_ precipitation increases sharply, leading to rapid rises in hardness and tensile strength.
2. The peak-aging condition is 150 °C for 48 h. This optimal T8 treatment yields excellent strength–ductility synergy: L-direction YS is 521 MPa, UTS is 541 MPa, and EL is 11.1%; LT-direction values are 486 MPa, 548 MPa, and 12.2%, respectively. Over-aging (96 h) reduces strength but increases corrosion susceptibility.
3. T_1_ phase evolution governs both mechanical and corrosion behaviors. The 3% pre-stretching creates dislocation networks for T_1_ nucleation. At 150 °C, the T_1_ size grows from ~66.4 nm (36 h, under-aged) to ~80.2 nm (48 h, peak-aged), then coarsens to ~117.8 nm (96 h, over-aged), correlating with the monotonic rise in strength and hardness. Corrosion in Al–Li alloys is mainly controlled by galvanic cells between the matrix and precipitates, and its mode follows a U-shaped path as a function of aging time: severe IGC at 0.5 h; transition to pitting corrosion with the shallowest attack develops at 16 h due to the breakdown of continuous GB precipitates and the appearance of intragranular ones; deeper pitting is observed at 48 h with a higher density of intragranular T_1_ phases; and severe IGC recurrence at 96 h due to the widening of PFZs. In contrast, at a lower aging temperature (120 °C), insufficient T_1_ nucleation leads to persistent IGC without pitting. Thus, the evolution of T_1_ size, GB precipitate continuity, and PFZ width influences both mechanical performance and localized corrosion resistance.

In summary, the 2060 Al–Li alloy under T8 treatment at 150 °C for 48 h achieves high strength and a pitting-dominated corrosion mode, during which controlled T_1_ precipitation and tailored GB morphology play a key role in balancing mechanical and corrosion performance in this alloy.

## Figures and Tables

**Table 1:** Chemical composition of 2060 Al–Li alloy (wt. %).

Element	Cu	Li	Zn	Mg	Ag	Mn	Zr	Fe	Ti	Si	Al
Content	3.70	0.74	0.40	0.65	0.36	0.30	0.11	<0.10	<0.10	<0.10	Bal.

## Data Availability

The original contributions presented in this study are included in the article. Further inquiries can be directed to the corresponding author.

## Appendix A

**Table A1:** Main corrosion reactions of the 2060 Al–Li alloy in NaCl + H_2_O_2_ solution.

Type of Reactions	Chemical Equation
Anodic reaction	Al → Al_3+_ + 3e^−^
Cathodic reaction	O_2v_ + 2H_2_O + 4e^−^ → 4OH^−^
Hydrolysis and pit acidification	Al^3+^ + 3H_2_O → Al(OH)_3_ + 3H^+^
